# Rising of antimalarial drug resistance *Pfmdr1* N86Y and *Pfcrt* K76T gene in Ethiopia: a systematic review and meta-analysis

**DOI:** 10.1016/j.jgeb.2026.100691

**Published:** 2026-04-02

**Authors:** Temesgen Mitiku yeshanew, Betelhem Abebe Begashaw, Gemechis Waktole Bayisa, Birhan Getie, Nega Birhane

**Affiliations:** a Department of Biotechnology, Dambi Dollo University, P.O. Box 260, Dambi Dollo, Ethiopia; b Department of Medical Biotechnology, Institute of Biotechnology, University of Gondar, P.O. Box 196, Gondar, Ethiopia

**Keywords:** Malaria, *Plasmodium falciparum*, Mutations, Drug resistance, *Pfmdr1*, *Pfcrt*, Ethiopia

## Abstract

**Background:**

In sub-Saharan Africa, including Ethiopia, malaria continues to pose a significant public health risk. Efforts to control the disease are complicated by emerging resistance to current drugs and concerns about the sustained effectiveness of antimalarial medications. The purpose of this systematic review and *meta*-analysis is to determine the nationwide prevalence of the *Pfcrt* K76T and *Pfmdr1* N86Y genetic mutations in Ethiopia.

**Methods:**

We queried multiple sources for literature, including the Google Scholar, Cochrane Library, Scopus, Web of Science, and PubMed/MEDLINE databases. Final calculations of overall prevalence were displayed in a forest plot. We subsequently conducted a subgroup analysis to determine any variations or differences among the included studies. Publication bias was assessed visually with funnel plots. The entirety of the statistical evaluation was completed using STATA software (version 16).

**Results:**

Among the 1,843 initially identified studies, twelve full-text articles met the inclusion criteria and were included in the analysis. The pooled prevalence estimates for *Pfcrt* K76T and *Pfmdr1* N86Y were 75% (CI 62–88) and 24% (CI 7–42), respectively. In a subgroup analysis of studies published between 2021–2025 the pooled prevalence of *Pfcrt* K76T and *Pfmdr1* N86Y was 77% and 14%, respectively. In contrast, studies published from 2006 to 2019 revealed different trends, with a lower pooled prevalence of *Pfcrt* K76T at 74% and *Pfmdr1* N86Y at 29%

**Conclusions:**

This systematic review and *meta*-analysis demonstrated a significant prevalence of the *Pfcrt* K76T and *Pfmdr1* N86Y gene mutation in Ethiopia. Consequently, there is a pressing need to enhance prevention and control measures and implement new strategies to address this issue.

## 1 Background

The burden of malaria on global health is substantial, causing high rates of morbidity and mortality. This threat is particularly acute in Africa, where the sub-Saharan region bears the heaviest consequences of the disease [1], [2]. According to the World Health Organization (WHO) latest World Malaria Report, there were an estimated 263 million cases and 597,000 malaria deaths worldwide in 2023 [3]. Approximately 95% of the estimated 597,000 global malaria deaths in 2023 were reported in the African Region by the WHO [3]. Among *Plasmodium* species, *Plasmodium falciparum* is a significant concern in Africa and is responsible for approximately 90% of global cases and deaths [4].

Numerous challenges, including funding shortages and parasite resistance, impede malaria eradication effort [5]). The evolution of malaria is influenced by genetic, host, parasite, vector, pharmacogenetic, environmental, and epigenetic factors [6], [7]. Genetic factors in parasites include mutations at drug target sites or an increase in the copy number of genes associated with drug targets, which may impede the efficacy of antimalarial drugs [8]. Genetic differences in a host's ability to metabolize specific antimalarial drugs influence the concentration of the drug in the blood. This variation in drug plasma levels can cause inconsistent treatment outcomes and contribute to the evolution or spread of drug-resistant parasites [9], [10]. The overall development of antimalarial drug resistance is also influenced by several parasite- and treatment-related factors, such as the parasite's mutation rate, the patient's parasite burden, the drug's effectiveness, and issues with treatment adherence and compliance with malaria guidelines Shibeshi et al., 2020; Organization, Shibeshi et al., 2020; Organization, [11], [12].

Numerous scientific investigations have pinpointed structural variants (such as duplications, amplifications, and copy number variations) and single nucleotide polymorphisms (SNPs) that are linked to resistance against antimalarial drugs like chloroquine, sulfadoxine-pyrimethamine (SP), and artemisinin [13], [14]. These key variations occur in *Plasmodium falciparum* genes, specifically the chloroquine resistance transporter gene (*Pfcrt*), the artemisinin resistance kelch 13 gene, the dihydrofolate reductase gene (*pfdhfr*), the dihydropteroate synthase gene (*Pfdhps*), and the multidrug resistance 1 gene (*Pfmdr1*) [15].

The primary cause of chloroquine resistance (CQR) in the deadly human malaria parasite, *Plasmodium falciparum*, is mutations found in the parasite's chloroquine resistance transporter gene (*Pfcrt*) [16]). Specifically, the K76T polymorphism within the *Pfcrt* gene, where the amino acid Lysine (K) is replaced by Threonine (T) at position 76, is widely recognized as a molecular marker for CQR and is closely linked to treatment failure [17]. However, research indicates that the K76T mutation does not act in isolation; it interacts with other *Pfcrt* mutations at positions 72, 73, 74, and 75 [18], [19]. Consequently, CQR strains of *P. falciparum* may exhibit triple CVIET mutations, primarily found in Southeast Asia and Africa, or double SVMNT mutations, which are prevalent in South America [20], [21].

Sulfadoxine-pyrimethamine (SP) became a key treatment for malaria following the extensive resistance observed against chloroquine [22], [23]. This therapeutic approach was later substituted by artemisinin combination therapies due to the increasing frequency of *P. falciparum* mutant alleles. Mutations in the *P. falciparum* genes dihydrofolate reductase (*Pfdhfr*), [24], and dihydropteroate synthase *(Pfdhps)* [25] genes specifically at codons 51, 59, 108, and 164 of *Pfdhfr*, and 437, 540, and 581 of *Pfdhps*, are correlated with SP treatment failure [26]. Furthermore, the parasite's *Kelch13* propeller gene has been identified as being associated with resistance to the current front-line antimalarial, artemisinin [27]. While *kelch13* mutations are linked to reduced potency in vitro, therapeutic failure, and high prevalence, these novel mutations require further validation [28].

The *Pfmdr1* gene is known to harbor several documented polymorphisms, including N86Y, Y184F, S1034C, N1042D, and D1246Y. Of these, N86Y, Y184F, and D1246Y are the most commonly cited variations [15]. The N86Y mutation is considered the most significant because it modifies the protein's transport function and is strongly linked to high chloroquine (CQ) resistance [29], [30]. Intriguingly, this same mutation confers increased sensitivity to certain other antimalarials, specifically lumefantrine (LUM), mefloquine (MQ), and dihydroartemisinin [31]. A significant linkage disequilibrium has been observed between the K76T mutation in *Pfcrt*, which causes chloroquine resistance in *Plasmodium falciparum*, and the N86Y mutation in pfmdr1 isolates of the parasite [32].

In Ethiopia, similar to other countries with a high prevalence of malaria, the regular monitoring of molecular markers for antimalarial-drug resistance has not been consistently conducted to assess the effectiveness of treatment. This was primarily due to the limited capacity. However, researchers, particularly PhD students specializing in medical biotechnology, biotechnology or any health-related field, possess expertise in conducting research on molecular markers related to antimalarial resistance. Their goal was to collect data on the prevalence of mutations found in the *Pfcrt* and *Pfmdr1* genes [20], [33]. The main goal of this systematic review and *meta*-analysis was to determine the pooled prevalence of the *Pfcrt* K76T and *Pfmdr1* N86Y mutations in *Plasmodium falciparum* across Ethiopia and to map their regional and temporal variations. Ultimately, this study offers a national evaluation of the accelerating resistance to crucial antimalarial drugs.

## 2 Methods

The methodology for this study adhered to the guidelines established by the Preferred Reporting Items for Systematic Reviews and 10.13039/100019827Meta-Analyses (PRISMA) [34].

### 2.1 Systematic review and *meta*-analysis question

In regions of Ethiopia where malaria is common, what proportion of *Plasmodium falciparum* parasites exhibit the *Pfcrt* K76T and *Pfmdr1* N86Y resistance-associated alleles?

### 2.2 Systematic review and *meta*-analysis objectives

This systematic review and *meta*-analysis will establish the frequency (or prevalence) of two key drug-resistance markers in *P. falciparum* parasites found in Ethiopia: the *Pfcrt* K76T allele (related to chloroquine resistance) and the *Pfmdr1* N86Y allele (associated with multidrug resistance). Additionally, the study seeks to identify any changes or trends in the prevalence of both the *Pfcrt* K76T and *Pfmdr1* N86Y alleles over time within malaria-affected areas of the country.

### 2.3 Registration of the protocol

The complete protocol for this systematic review and *meta*-analysis was filed and is publicly available on the International Prospective Register of Systematic Reviews (PROSPERO). The registration ID is CRD42024583008 (accessible at: https://www.crd.york.ac.uk/prospero/record_email.php).

### 2.4 Study Area

Ethiopia is a large nation, spanning 1.1 million square kilometers, defined by its extremely diverse topography, with elevations ranging from 110 m below sea level to 4,550 m above sea level. Its climate is predominantly a tropical monsoon, which naturally divides the country into three distinct agroecological regions: the lowlands, midlands, and highlands. These regions are characterized by specific temperature and rainfall patterns. The highlands have the coolest climate, with average annual temperatures between 10°C and 16°C, and they receive the highest rainfall, varying from 500 mm to over 2,000 mm annually. The midlands have more moderate temperatures, averaging 16° C to 29° C, while the lowlands are the hottest region, with temperatures from 23° C to 33° C, and significantly lower annual rainfall, ranging from 300 mm to 700 mm [35]. With a population currently estimated at over 120 million, a significant majority approximately 68% of Ethiopians live in areas where they are at risk of malaria [36].

### 2.5 Literature search strategy

The primary objective of this systematic review and *meta*-analysis was to establish the prevalence of the *Pfcrt* and *Pfmdr1* drug resistance genes within Ethiopia. We adhered to the PRISMA (Preferred Reporting Items for Systematic Reviews and Meta-analysis) guidelines for our search strategy, selection of publications, and reporting of the findings [37]. A comprehensive electronic search was performed across major databases, including PubMed/MEDLINE, Web of Science, Scopus, the Cochrane Library, and Google Scholar. The search utilized various terms combined with Boolean operators (e.g., OR, AND). Typical combinations of keywords included: *Plasmodium falciparum*, drug resistance gene, *Pfcrt*, *Pfmdr1*, prevalence, malaria, and Ethiopia. To supplement the electronic search, we also conducted manual searches using Google and thoroughly reviewed the reference lists of all articles selected for inclusion to identify any relevant supplementary literature.

### 2.6 Eligibility criteria

A comprehensive overview of the participants, interventions, comparators, and outcomes evaluated, along with the categories of studies incorporated on the basis of the PICOS criteria [38], is presented in Table 1. We did not place any time restrictions on our searches; however, all eligible studies were published between 2006 and 2025.

**Table 1:** PICOS strategy and eligibility criteria.

**PICOS Strategy**	**Inclusion criteria**	**Exclusion criteria**
P:Population	i. Human participants of any age, gender, or clinical status infected with *Plasmodium falciparum* in Ethiopia. ii. *P. falciparum* isolates (from blood samples) collected within Ethiopia.	i. Studies focusing exclusively on other *Plasmodium* species. ii. Studies where the geographic origin of the parasite isolate is outside of Ethiopia or cannot be confirmed.
I: Intervention	i. Molecular genotyping for the presence of the *Pfcrt* K76T and *Pfmdr1* N86Y gene mutations.ii. Studies that report the prevalence (frequency) of these mutations in the studied population.	i. Studies that only report phenotypic drug resistance (e.g., in vitro assays, therapeutic efficacy trials) without accompanying genotypic data for the specified mutations.ii. Studies that only investigate other resistance markers (e.g., *Pfkelch13, Pfdhfr, Pfdhps*) without data on *Pfcrt* K76T and *Pfmdr1* N86Y.
C:Comparison	i. The prevalence of the mutant genotype 76 T was compared to the prevalence of 86Y within the same study populations ii. Subgroup analysis was compare prevalence rates within year of study	i. Studies that do not allow for the calculation of the number of mutant and wild-type alleles or genotypes
O: Outcome	i. Pooled prevalence of the *Pfcrt* K76T mutation among *P. falciparum* isolates in Ethiopia ii. Pooled prevalence of the *Pfmdr1* N86Y mutation among *P. falciparum* isolates in Ethiopia.	i. Studies that do not report quantitative data on the frequency or prevalence of the specified genotypes. ii. Studies with outcomes only reported as narrative descriptions without extractable numerical data.
S: Study design	Cross-sectional studies	i. Case reports, case series (<10 samples), editorials, letters, and narrative reviews.ii. In vitro or animal studies (nonhuman data) . iii. Studies with a sample size of less than 10*P. falciparum* confirmed isolates (to avoid small study bias). Iv. Full text not available after attempting to contact authors.

### 2.7 Outcome measurement

The central focus of this research was to quantify the prevalence of antimalarial drug resistance in Ethiopia, specifically by examining two key genetic markers: the *Pfcrt* gene mutation at codon 76 and the *Pfmdr1* gene mutation at codon 86.

To calculate this estimate, we utilized the metan prevalence standard error command after first computing the individual study prevalence rates and their corresponding standard errors. The necessary data for this outcome was extracted and organized into two-by-two tables within a Microsoft Excel spreadsheet.

### 2.8 Article selection and data extraction

Initially, all retrieved articles were imported into EndNote X7 software to identify and remove duplicate files. The authors then independently screened the remaining articles, reviewing titles, abstracts, and full texts against the established inclusion criteria. Next, a comprehensive data extraction form was developed using a Microsoft spreadsheet to systematically gather essential details from the full-text articles. This spreadsheet captured information such as the first author's name, publication year, region (province), geographic location, study group, study design, sample size, sampling technique, diagnostic method, and the total number of positive findings (both overall and species-specific). Key findings relevant to the systematic review were also extracted for qualitative analysis.

To ensure accuracy, three investigators checked the extracted data for consistency, resolving any discrepancies through discussion. Finally, the complete data set was formally reviewed and approved by Birhan Getie, Betelhem Abebe, and Nega Birhane.

### 2.9 Assessment of risk of bias

The potential risk of bias for every study included was evaluated independently by four of the authors (TM, BA, BG, GW and NB). This assessment utilized the Prevalence Critical Appraisal Instrument, a tool specifically designed for systematic reviews focusing on prevalence questions, as detailed by Munn et al. [39]. This instrument assesses the methodological rigor of studies that report prevalence data across ten critical appraisal criteria. These criteria include evaluating: the representativeness of the sample relative to the target population, the suitability of participant recruitment methods, the adequacy of the sample size, the thoroughness of the description of subjects and setting, the completeness of the sample coverage, the objectivity and standardization of the condition's measurement, the reliability of that measurement, the appropriateness of the statistical methods used, the identification and handling of potential confounders, subgroups, or differences, and the use of objective criteria to define subpopulations.

### 2.10 Data synthesis and analysis

Data from each original study were extracted and compiled in a Microsoft Excel spreadsheet before being exported to STATA (Windows version 16) for statistical analysis. For the initial outcome, the prevalence and its standard error were calculated for each study using STATA's “generate” command. Similarly, the logarithm and standard error of the Odds Ratio (OR) were determined for the second outcome variable. The combined magnitude of *P.falciparum Pfcrt* and *Pfmdr1* drug resistance gene prevalence is visually presented in a forest plot. Heterogeneity between studies was evaluated using Cochran's Q test (reporting the p-value) and the I [2]statistic. A p-value ≤ 0.05 for Cochran's Q test indicated statistically significant heterogeneity. The I [2] values of 0%, 25%, 50%, and 75% were interpreted as no, low, moderate, and high heterogeneity, respectively [40]. Due to the observed high heterogeneity, a random effects model was implemented to estimate the pooled prevalence of the *pfcrt* and *pfmdr1* drug resistance genes. Subgroup analysis was performed using categorical variables to investigate potential study differences. Additionally, *meta*-regression was conducted based on the sample size and publication year. Publication bias was assessed visually using a funnel plot and statistically using Egger's test. Finally, the trim-and-fill method was utilized to adjust for any statistically significant publication bias identified.

### 2.11 Study quality assessment

To ensure rigor, all authors independently applied the JBI quality assessment tool for prevalence studies [41] to evaluate the included studies. The nine items on the JBI checklist were scored as follows: 2 points for “Yes,” 1 point for “No,” and 0 points for “Unclear” or “Not applicable.” The total score for each article ranged from 0 to 18. Studies were then grouped by quality: high quality (14–18 points), moderate quality (9–13 points), or poor quality (0–8 points). Only articles deemed high quality were ultimately included in the synthesis, with all quality assessment scores presented in a table for comparative analysis.

### 2.12 Ethical consideration

These investigations were conducted entirely in accordance with PRISMA guidelines [42]. Approval from the institutional review board or ethics committee was not necessary because this was a systematic review and *meta*-analysis.

## 3 Results

### 3.1 Study selection

**Fig. 1:**
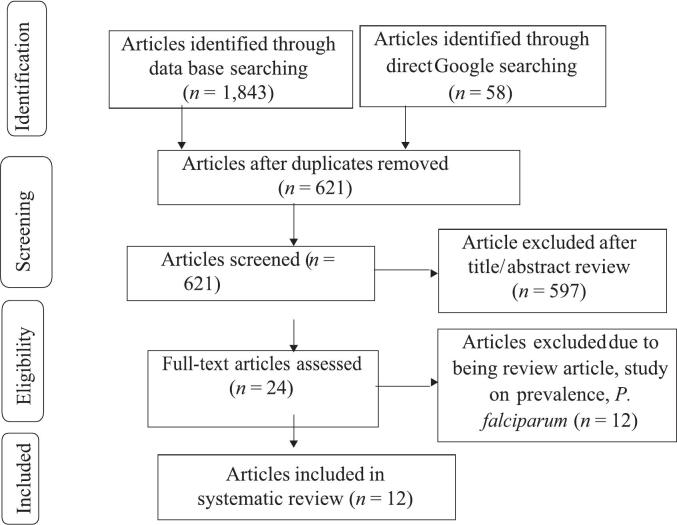
PRISMA flow diagram showing the study selection process, 2025.

The prevalence of the *Pfcrt* gene at codon 76 and the *Pfmdr1* gene at codon 86 associated with antimalarial drug resistance in Ethiopia yielded a total of 1,893 articles. A total of 1,272 of these papers were removed because they were redundant or unrelated to the research. A total of 597 additional papers were removed after the titles and abstracts were screened. Overall, 24 studies were found to be eligible. Of those, 12 studies were removed for a variety of reasons, including not focusing on the prevalence of drug resistance, not being conducted in Ethiopia, not having enough data, or failing to disclose the pertinent outcome. Ultimately, this analysis included 12 studies (Fig. 1). Among the studies included, the study conducted in the Oromia and Gambella region, Ethiopia, had the smallest sample size, with 27 participants [43]. A study carried out in the Benishangul-Gumuz region of Ethiopia had 1,147 participants, which was the second-highest number among the included studies [44]. Most of the studies included in this analysis were conducted in the Oromia region [20], [33], [43], [45], [46], [47], [48], followed by southern Ethiopia [44], [48], [49]. Other datasets were obtained from studies conducted in the Amhara [45], Benishangul Gumuz [46], [50], and Gambella [43], [46] regions. The studies conducted in Amhara, Gambella, Oromia, SNNP, and Somali had the largest sample size of 1,199 [51] (Table 2). However, no studies were reported from the remaining Ethiopian regions. The quality of every study incorporated into this systematic review and *meta*-analysis was evaluated using the JBI quality assessment tool. Collectively, these studies contained 2,810 *Plasmodium falciparum*-positive samples.

**Table 2:** Included studies in the *meta*-analysis of the prevalence of the *pfcrt* and *pfmdr1* drug resistance genes in *Plasmodium falciparum* in Ethiopia.

**Author/year**	**Region**	**Study group**	**Method**	**Sample**	**Positive sample**	**Prevalence *Pfmdr1* N86Y**	**Prevalence *Pfcrt* K76T**
Addimas et al. 2015	Amhara	Suspected	SYBR Green I	169	133	133	73
Lo et al., 2017	Amhara& Oromia	Suspected	Sequencing	430	226	3	116
Golassa,et al., 2015	Oromia & Gambella	Positive	PCR-RFLP	152	152	14	146
Hailemeskel et al*.*, 2019	Gambella, Benishangul-Gumuz and Oromia	Positive	PCR-RFLP	183	183	10	129
Abera et al., 2021	Oromia	Positive	Whole-genome analysis	34	34	14	34
Schunk et al., 2006	SNNP	Positive	PCR-RFLP	100	69	45	69
Mekonnen *et al*., 2014	SNNP and Oromia	suspected	Sequencing	410	195	33	40
Hassen et al., 2022	Oromia	Positive	PCR-RFLP	121	121	0	76
Mula et al., 2011	SNNP	suspected	PCR-RFLP	1,147	76	25	76
Tadele et al., 2023	Benishangul-Gumuz	Positive	HRM	230	225	42	152
Heuchert et al., 2015	Oromia	Positive	PCR-RFLP	338	197	3	153
Brhane 2025	Amhara, Gambella, Oromia, SNNP and Somali	Positive	Sequencing	1199	1199	12	896

### 3.2 Assessment of publication bias

Publication bias was assessed both subjectively and objectively. Subjectively, funnel plots were used to visually evaluate the presence of publication bias. Each point on the funnel plots represents a separate study, and an asymmetrical distribution indicates the presence of publication bias [52]. The funnel plots were slightly asymmetrical in all the cases (Fig. 4). However, to objectively evaluate the evidence from the funnel plots, Egger's weighted regression was used. According to the symmetry assumption, there was publication bias in the *Pfcrt* (τ2 = 0.05) and *Pfmdr1* (τ2 = 0.1) resistance pooled prevalence estimates.

### 3.3 Pooled prevalence rates of *Pfcrt* K76T and *Pfmdr1* N86Y mutations

**Fig. 2:**
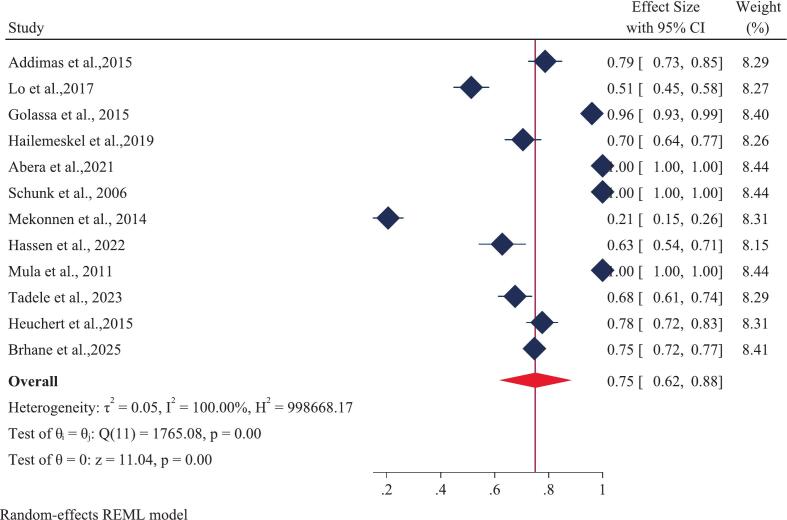
Forest plot displaying the pooled prevalence of the *Pfcrt* K76T gene mutation in Ethiopia: 2025.

**Fig. 3:**
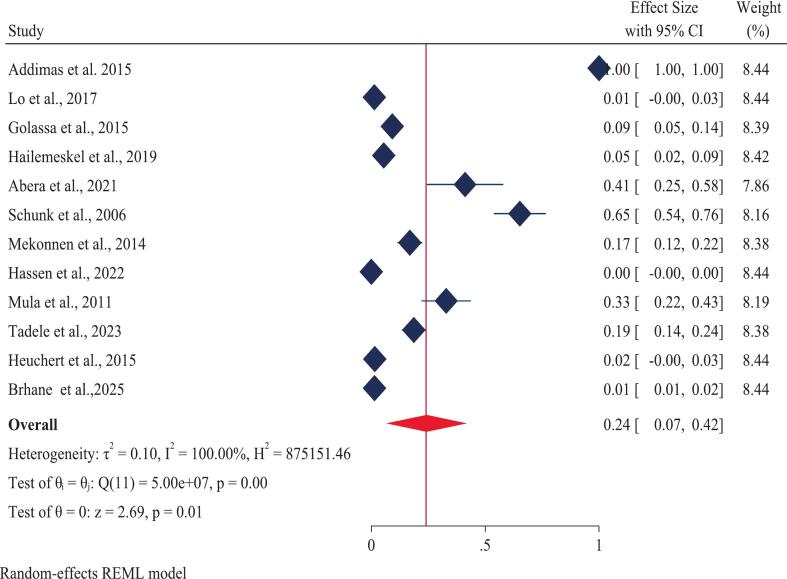
Forest plot displaying the pooled prevalence of the *Pfmdr1* N86Y gene mutation in Ethiopia: 2025.

A total of twelve studies, encompassing 2,810 *Plasmodium falciparum*-positive samples, were analyzed. The overall (pooled) prevalence of the *Pfcrt* K76T mutation in Ethiopia was estimated at 75% (CI 62–88) (Fig. 2). This result demonstrated significant heterogeneity across the studies (I [2] = 100%, p-value = 0.00). Examining individual study results, the highest reported prevalence of *Pfcrt* K76T (100%) was found in three separate studies: one covering Oromia and Gambella [20], one solely in Oromia [53] and another in SNNP [54]. Conversely, the lowest prevalence of *Pfcrt* K76T (21%) was documented in studies from the SNNP and Oromia regions [55]. For the *Pfmdr1* N86Y mutation, the pooled prevalence using a random-effects model was 24% (CI 7–42) (Fig. 3), also exhibiting substantial heterogeneity (I [2] = 100%, p-value = 0.00). Regionally, the prevalence of *Pfmdr1* N86Y varied widely, ranging from a maximum of 100% in the Amhara region to a minimum of 0.00% in the Oromia region.

**Fig. 4:**
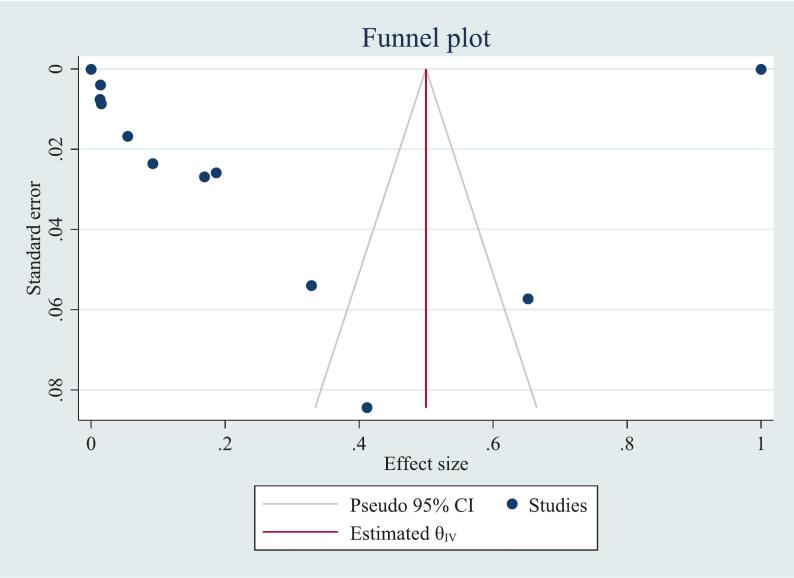
Funnel plot of the increasing prevalence of the *Pfcrt* K76T and *Pfmdr1* N86Y gene mutations in Ethiopia: Systematic review and *meta*-analysis, 2025.

### 3.4 Subgroup analysis the prevalence of the *Pfcrt* K76T and *Pfmdr1* N86Y mutations

Significant heterogeneity was observed across the included studies, with the Inverse of Variance (I [2] statistic reaching 100% for both the *Pfcrt* K76T and *Pfmdr1* N86Y drug resistance markers. Consequently, a subgroup analysis categorized by publication year was performed to explore the reasons for this wide variation.

**Fig. 5:**
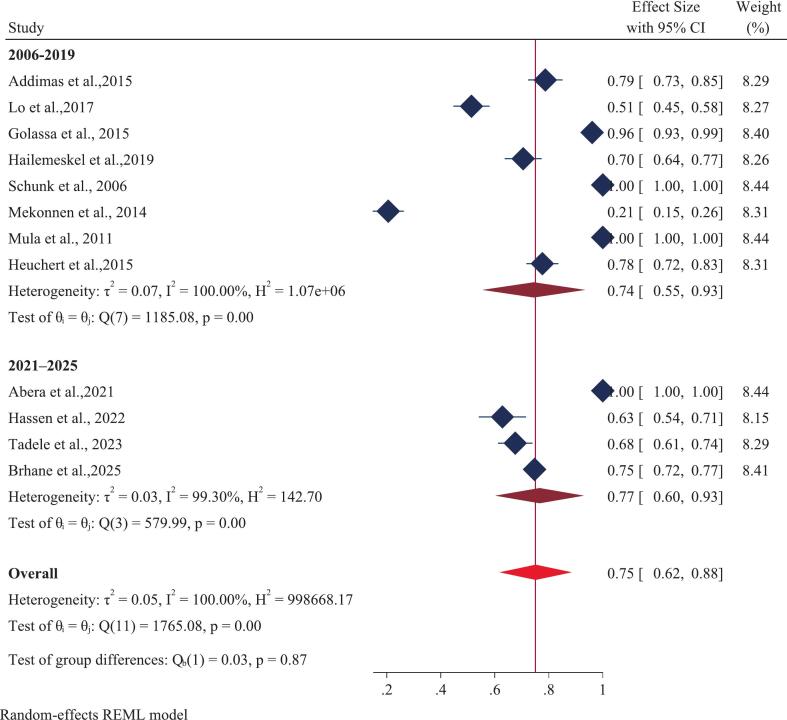
Forest plot displaying subgroup analysis based on year of publication of prevalence of *Pfcrt* K76T gene mutation in Ethiopia: 2025.

**Fig. 6:**
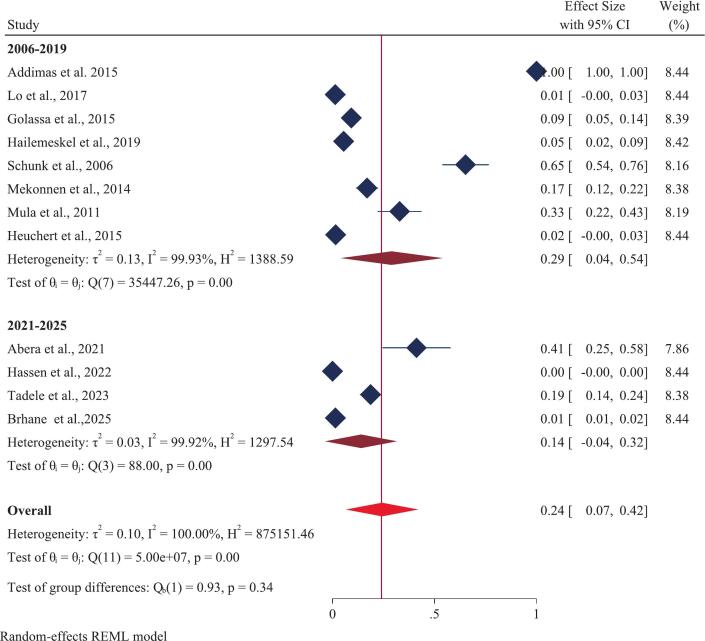
Forest plot displaying subgroup analysis based on year of publication of prevalence of *Pfmdr1* N86Y gene mutation in Ethiopia: 2025.

This *meta*-analysis revealed that the publication date significantly affected the prevalence of both the K76T and N86Y markers. In a subgroup analysis of studies published between 2021 and 2025, the pooled prevalence of *Pfcrt* K76T was 77% (95% CI, 60–93) (Fig. 5), while the prevalence of *Pfmdr1* N86Y was 14% (95% CI, −4–32) (Fig. 6). In contrast, studies published from 2006 to 2019 showed different trends, with a lower pooled prevalence of *Pfcrt* K76T at 74% (95% CI, 55–93) (Fig. 5) and a prevalence of *Pfmdr1* N86Y at 29% (95% CI, 4–54) (Fig. 6).

## 4 Discussion

In this systematic review and *meta*-analysis, the pooled prevalence of the *Pfcrt* K76T gene mutation reached 75%. This outcome is consistent with reports indicating high antimalarial resistance in Nigeria (69.6%) [56] and India (78%) [57]. Conversely, this 75% figure surpasses the prevalence rates documented in several other studies, such as those from India (2.1%) [58], Madagascar (2.6%) [59], sub-Saharan African countries (26.9%) (26.9%) [60] and Senegal (37.2%) (37.2%) [61]. This observation pertains to self-prescribed medications, the uncontrolled use of herbal remedies, ongoing pressure to use drugs, and the acquisition of substandard antimalarial drugs from unauthorized vendors. Most individuals who obtain antimalarial medicine from these vendors are unaware of the types of antimalarial drugs being sold to them. This increase can be attributed to several factors, including the timing of policy changes, the ongoing use of chloroquine as a chemoprophylactic in children with sickle cell disease, and the continued use of chloroquine in combination with other antimalarials [62].

Conversely, this finding is lower than rates reported in Uganda (98%) [63], and the Solomon Islands (98.4%) [64]. The increase in the mutation rate could be attributed to the continued use of chloroquine in combination with other antimalarial drugs [62]. The observed phenomenon may stem from the change in drug policy: chloroquine was substituted as the first-line therapy by artemisinin-based combination treatments, which subsequently drove down the prevalence of the *Pfcrt* K76T mutation in the mentioned regions [65]. The aggregated prevalence of the *Pfcrt* K76T gene mutation rose markedly from 28% from 2006 to 2019 to 77% from 2021‒2025, indicating the rapid reappearance of parasites that possess this resistance marker.

The pooled prevalence of the *Pfmdr1* N86Y gene mutation was 24%. This finding aligns with reports from China (19.9%) [66], another study in China (22.2%) [67], sub-Saharan African countries (25.9%) [60], East Africa 32.4% [68], and Nepal (33%) [69]. The observed similarities may be ascribed to the comparable Plasmodium species concerning their susceptibility to antimalarial drugs, as well as the analogous malaria management and treatment practices prevalent in these regions.

In contrast to the findings above, the Ethiopian prevalence was lower than that observed in countries like East Africa (43.8%) [70], Cameroon (62.4%) [71], Sudan (53.6%) [72], India (54%) [57], the Democratic Republic of the Congo (66.7%) [73], and the Solomon Islands (98.4%) [62]. Potential reasons for this variation include differences in antimalarial drug use, dosing practices for active malaria, and the intrinsic genetic and metabolic adaptability of the parasite [74]. However, it is noteworthy that the prevalence in this study still exceeded rates reported in India (3.4%) [58], Myanmar (2.5%) [17], and Senegal (16.6%) [61]. The prevalence of the *Pfmdr1* N86Y gene mutation has decreased from 74% (2006–2019) to 14% (2021–2025), reflecting a notable reduction in the number of parasites that protect this particular resistance marker.

## 5 Conclusion

The findings from this systematic review and *meta*-analysis specifically the rising prevalence of the *Pfcrt* K76T and *Pfmdr1* N86Y gene mutations in Ethiopia underscore the high burden of antimalarial drug resistance. This indicates that drug-resistant malaria parasites are becoming more common, creating a major public health problem that requires a varied approach. The surge in the *Pfcrt* K76T mutation is a key contributor to the rapid spread of resistance. This situation highlights the indispensable need for sustained surveillance and dedicated research to discover new antimalarial drugs and develop countermeasures against drug resistance. Ultimately, improved monitoring of the *Pfcrt* K76T and *Pfmdr1* N86Y mutations, as well as efforts to identify and limit drug-resistant strains, are crucial. This includes utilizing drug combinations, optimizing existing treatments through better diagnostics, enhancing surveillance, and exploring non-drug approaches such as vaccines and vector control. Key elements of current and future strategies involve combining drugs with different resistance mechanisms, reintroducing older medications, and implementing a test before treat policy with appropriate diagnostics.

### 5.1 Strengths of this review

This study has several strengths. First, the researchers used multiple databases for their article search, both through manual and electronic methods. This comprehensive approach ensures that a wide range of relevant studies are considered. Additionally, the material was abstracted in a predetermined manner by three separate reviewers, which helps minimize errors and increases the reliability of the findings. Furthermore, the *meta*-analysis included studies from various regions of the country.

### 5.2 Limitations

The pooled prevalence of *Pfcrt* and *Pfmdr1* drug resistance gene mutations may be influenced by the inclusion of articles published exclusively in English. As the studies included in the analysis were of a laboratory-based cross-sectional design, the outcome variable could have been influenced by other confounding variables. Furthermore, the distribution of the included studies was not proportional across the country. More than one-third of the studies were derived from the Oromia region, whereas no studies were obtained from the Tigre and Afar regions.

**Consent to publish declaration:**

Not applicable.

**Ethics and consent to participate declarations:**

Not applicable.

**Authors’ contributions**

TM participated in the process of searching for and selecting articles. BA was involved in reviewing the studies and extracting the data. TM conducted the statistical analysis and interpreted the data. All the authors collaborated to prepare the draft manuscript; BA, BG, GW, and NB also made revisions. TM completed the final version of the manuscript and submitted it to the journal. All the authors have read and approved the final manuscript before submission.

## Funding declaration

The authors received no financial support for the research and/or authorship of this article.

## CRediT authorship contribution statement

**Temesgen Mitiku yeshanew:** Writing – review & editing, Writing – original draft, Software, Methodology, Investigation, Formal analysis, Data curation, Conceptualization. **Betelhem Abebe Begashaw:** Supervision, Resources, Conceptualization. **Gemechis Waktole Bayisa:** Data curation, Supervision, Writing – review & editing. **Birhan Getie:** Visualization, Validation, Resources. **Nega Birhane:** Conceptualization.

## Declaration of competing interest

The authors declare that they have no known competing financial interests or personal relationships that could have appeared to influence the work reported in this paper.
